# Disseminated necrotizing leukoencephalopathy eight months after alemtuzumab treatment for multiple sclerosis

**DOI:** 10.1186/s40478-016-0352-1

**Published:** 2016-08-08

**Authors:** Imke Metz, Peter Rieckmann, Boris-Alexander Kallmann, Wolfgang Brück

**Affiliations:** 1 Department of Neuropathology, University Medical Center Göttingen, Georg-August-University, Robert-Koch-Str. 40, 37075 Göttingen, Germany; 2 Department of Neurology, Sozialstiftung Bamberg Hospital, Buger Str. 80, 96049 Bamberg, Germany; 3 Neurological Practice Kallmann, Kärntenstr. 2, 96052 Bamberg, Germany

## To the editor

We report a case of disseminated necrotizing leukoencephalopathy (DNL) occurring after alemtuzumab treatment for multiple sclerosis (MS). A 33 year-old female patient with a 14-year history of highly active, relapsing-remitting MS despite treatment with interferons, natalizumab and fingolimod was administered 12 mg of alemtuzumab for five consecutive days. Alemtuzumab is a humanized monoclonal antibody directed against the CD52 antigen that significantly reduces relapses, MRI activity and the rate of sustained accumulation of disability [1]. Autoimmune disorders such as autoimmune thyroid disease occur in up to 28 % of patients after alemtuzumab therapy and are associated with the phase of immune reconstitution [2, 3]. In addition, respiratory infections are well known side effects observed in 60 % of patients [2]. Eight months after treatment, during a phase of clinically stable MS, the patient was admitted to the hospital due to anemia with low hemoglobin which was observed in her for the first time, and warm autoimmune hemolytic anemia was diagnosed. The hemoglobin dropped as low as 2.4 g/dl, requiring therapy with corticosteroids, intravenous immunoglobulins, plasma separation, cyclophosphamide and erythrocyte substitution. The anemia could not be stabilized. A lower respiratory tract infection with consecutive septic shock developed. The patient lost consciousness and required intubation. No focal neurological deficits were documented, and due to the fulminant disease course no MRI was performed. The patient died six days after admission and a brain autopsy was performed.

**Fig. 1:**
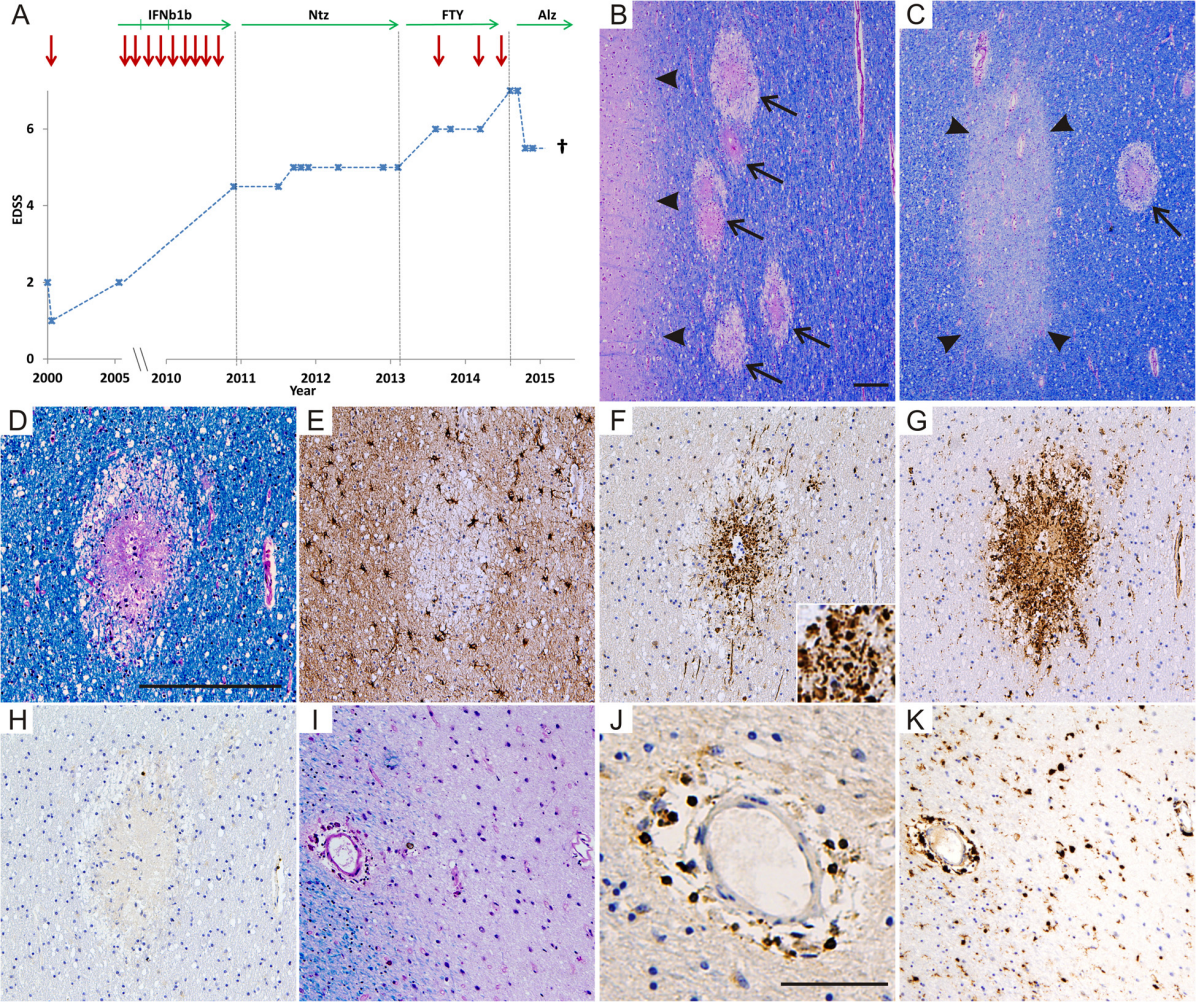
Clinical course and histopathology of DNL as well as MS lesions. The clinical course is illustrated in (**a**) with the expanded disability status scale (EDSS, score ranging from 0 to 10 with higher numbers indicating more severe disability), relapses (red arrows) and MS-specific treatment indicated over time. Clinical stability was achieved with natalizumab (Ntz) and alemtuzumab (Alz), but numerous relapses occurred with interferon beta-1b (IFNb1b) and fingolimod (FTY) treatment. Eight months after alemtuzumab infusions were given, DNL developed (**b**-**h**), characterized by multifocal disseminated necrotic lesions within the white matter (arrows in **b** and **c**, LFB/PAS), but not grey matter (arrowheads in **b**). In addition to DNL, typical MS lesions were present (arrowheads in C indicate remyelinated MS lesion). DNL lesions were characterized by acute and complete tissue destruction with a loss of myelin (**d**, LFB/PAS), oligodendrocytes and astrocytes (**e**, anti-GFAP), pronounced axonal damage (**f**, anti-APP, insert shows many APP-positive, brown stained damaged axons in higher magnification) and numerous macrophages (**g**, KiM1P) in the absence of lymphocytic infiltration (**h**, anti-CD3). In contrast, MS lesions showed demyelination (**i**, LFB/PAS) with axonal preservation (not shown), lymphocytic inflammation (**j**, anti-CD3) and a sparse infiltrate with macrophages (**k**, KiM1P). Scale bars represent 200 μm in **b** (valid for **b** and **c**) and **d** (valid for **d**-**i** and **k**) as well as 100 μm in **j**

Autopsy revealed, besides characteristic inactive MS lesions, numerous small, round, disseminated and necrotizing lesions in the supratentorial white matter, the cerebellum and the brain stem including the pons. These lesions were characterized by numerous axonal spheroids and a pronounced macrophage infiltrate in the absence of lymphocytes, consistent with DNL (Fig. 1) [4, 5].

Severe autoimmune hemolytic anemia has been reported after alemtuzumab therapy, and may be caused by an immune dysregulation with the development of autoimmunity following drug application [6, 7]. Paradoxically, alemtuzumab is also used to treat autoimmune hemolytic anemia [6, 8].

The etiology of DNL is still unknown. It was first described in patients with brain tumors treated with high-dose, methotrexate-based chemotherapy and whole brain irradiation [4, 5]. DNL has been found in immunosuppressed patients, including HIV patients [1]. Single case reports described DNL occurring with infectious diseases and sepsis, suggesting an excessive inflammatory response as etiologic factor [9]. Symptoms occur directly after therapy or many months later [4]. Thus, DNL in our patient may be related to the direct effects of alemtuzumab, e.g. immunosuppression, or it could be linked to side effects of alemtuzumab infusions such as the respiratory tract infection with sepsis or the warm hemolytic anemia and its immunosuppressive treatment. The autopsy showed acute necrotizing lesions, suggesting they developed simultaneously with the sepsis and hemolytic anemia.

Clinical symptoms of DNL include irritability, somnolence, rapidly progressive subcortical dementia and coma with fatal outcome. Neuroimaging may be normal in early stages and show white matter lesions, often calcified, in later stages [4]. The observed loss of consciousness in our patient may have been related to DNL. DNL can be difficult to recognize clinically and is thus easily overlooked.

In conclusion, the clinician should be aware of DNL as a possible direct or indirect side effect of alemtuzumab treatment in MS that is rare but severe, and has never before been described in the literature.
